# Successful Use of Heterologous CMV-Reactive T Lymphocyte to Treat Severe Refractory Cytomegalovirus (CMV) Infection in a Liver Transplanted Patient: Correlation of the Host Antiviral Immune Reconstitution with CMV Viral Load and CMV miRNome

**DOI:** 10.3390/microorganisms9040684

**Published:** 2021-03-26

**Authors:** Monica Miele, Alessia Gallo, Mariangela Di Bella, Francesca Timoneri, Floriana Barbera, Marco Sciveres, Silvia Riva, Paolo Grossi, Pier Giulio Conaldi

**Affiliations:** 1 Department of Research, IRCCS ISMETT (Istituto Mediterraneo per i Trapianti e Terapie ad Alta Specializzazione), Via E. Tricomi 5, 90127 Palermo, Italy; mmiele@ismett.edu (M.M.); madibella@ismett.edu (M.D.B.); ftimoneri@ismett.edu (F.T.); fbarbera@ismett.edu (F.B.); pgconaldi@ismett.edu (P.G.C.); 2 Fondazione Ri.MED, 90133 Palermo, Italy; 3 Pediatric Department, IRCCS ISMETT (Istituto Mediterraneo per i Trapianti e Terapie ad Alta Specializzazione), University of Pittsburgh Medical Center Italy, 90127 Palermo, Italy; msciveres@ismett.edu (M.S.); sriva@ismett.edu (S.R.); 4 Infectious Disease Unit, Department of Medicine and Surgery, University of Insubria, 21100 Varese, Italy; paolo.grossi@uninsubria.it

**Keywords:** solid organ transplant, cytomegalovirus infection, immunotherapy, allogenic T-cells, viral miRNAs

## Abstract

Cytomegalovirus (CMV) infection is the most significant viral infection in hosts with compromised immune systems as solid organ transplant patients. Despite significant progress being made in the prevention of CMV disease in these patients, further therapeutic strategies for CMV disease and for the CMV reactivation prevention are needed. Here, we describe the outcome of the infusion of in vitro expanded CMV-reactive T-cells, taken from a healthy CMV-seropositive donor, in a liver-transplanted recipient with a refractory recurrent CMV. In this particular case, adoptive transfer of allogenic CMV-reactive T-lymphocytes resulted in the clearance of CMV infection and resolution of the pathological manifestations of the patient. In the study we also investigated circulating miRNAs, both cellular and viral, as potential biomarkers during the course of CMV infection. The results indicate that the infusion of allogenic CMV-reactive T-cells can be an effective strategy to treat CMV infection recurrence when the generation of autologous virus specific T cell clones is not possible.

## 1. Introduction

Cytomegalovirus (CMV) primary infection or reactivation of latent viruses can cause severe disease [1] in immunocompromised patients. In particular, CMV-seronegative transplant recipients receiving a graft from a CMV seropositive donor are at high risk of CMV infection and CMV disease development [2]. Currently two alternative approaches for CMV prevention can be adopted, preemptive therapy and antiviral prophylaxis. Indeed, the prevention strategies may not be mutually exclusive and the choice should be based upon a risk–benefit evaluation, balancing drug-associated toxicity with the risk of developing CMV disease [3]. For pre-emptive therapy, patients are monitored regularly for CMV viral load, but the technical constraints of laboratory surveillance, and the lack of a widely applicable viral load threshold, reduce the practicality of this approach [4]. Pharmacological universal prophylaxis against CMV infection/reactivation is still based on dated antiviral compounds, mainly Ganciclovir (GCV) and Valganciclovir ((V)GCV), but also Cidofovir (CDF) and Foscarnet (FOS) for a minimum of one year after transplantation. Hematologic adverse events (i.e., bone marrow depression) are not uncommon using these drugs. Maribavir (MBV) and Brincidofovir (BDF) are two new promising anti-CMV drugs without myelosuppressive properties or renal toxic effects for which phase II and III trials are in progress to evaluate their safety and efficacy [5,6] while Letermovir (LMV) was approved in 2017 by the US Food and Drug Administration [7,8].

GCV and (V)GCV block viral replication through the inhibition of the viral DNA polymerase (UL54) activated by viral thymidine kinase (UL97). In the case of occurrence of GCV and (V)GCV-resistant CMV variants, Foscarnet and Cidofovir can be used as second-line/third line therapies, since they do not require activation by UL97, but they are related to higher toxicity [3,9].

However, every prolonged antiviral treatment (either first-line or second-line) may result in serious side-effects such as nephrotoxicity, myelosuppression and a delayed immune reconstitution that can favor the onset of late CMV disease [10,11]. Thus, drug toxicity and virus reactivation after the end of treatment are crucial issues of antiviral therapy of CMV infection, since [12] many patients require prolonged or repeated treatment [12,13].

In view of this, the restoration of adequate antiviral immunity due to adoptive T-cell therapy with CMV-specific T-cells, without occurrence of side effects, may exert a sustained control of refractory viral infections. Walter et al. [14] showed that pre-emptive adoptive transfer of CMV specific CD8^+^ T-clones protected the patients from virus-related complications in allogeneic stem cell grafts.

A standardized protocol for CMV-specific T cell generation is not established [15]. However, the clinical study from Einsele’s group [16] demonstrated that CMV-specific polyclonal T-cell infusion in GCV nonresponding CMV-infected patients was successful in five of seven recipients. This evidence suggests that specific immune reconstitution can represent a pivotal tool for long-term control of CMV replication [17,18].

In this report we describe the possible use of heterologous T-cells, pulsed with the viral proteins and expanded in vitro, to successfully reconstitute the CMV–specific CD8^+^ and CD4^+^ immune response in a solid organ transplant (SOT) patient.

Since CMV encodes for 26 miRNAs [19] that have been reported to regulate immune evasion [20] and virus replication [21] by interfering with the host immune system by regulating viral and cellular gene expression [22]. Very little is known about the expression profile of viral and cellular miRNAs in SOT patients. We analyzed the expression of cellular and CMV miRNome during a period of 18 months, which covers all the CMV infection/relapses phases, in order to provide novel data and correlations useful for following the viral transcriptional phase and to understand disease status.

### Case Presentation

The patient was a 16-year-old male (CMV seronegative at the time of transplant) who underwent liver transplantation for Primary Sclerosing Cholangitis from a 4/6 HLA-matched deceased and unrelated donor (CMV seropositive). Twenty-one days after the transplantation, the patient developed a symptomatic primary CMV infection (CMV-DNA 1,686,000 copies/mL) characterized by leukopenia (760 cells/μL) and interstitial pneumonia. After 20 days-therapy with GCV followed by one month with Valganciclovir (900 mg × 2 vv/day), CMV viral load decreased but a severe absolute leukopenia persisted (1160 cell/μL). Two weeks later, the infection recurred (CMV DNA 193,300 copies/mL) with manifestation of alveolar pneumonitis associated with a worsening of the clinical status (weight lost and sleeping status). The patient was treated with Valganciclovir, 900 mg × 2 vv/day, resulting in a decrease of CMV DNAemia. One month later, another increase of CMV-DNA (116,829 copies/mL) was observed and a third cycle (one month) of antiviral therapy was started and associated with the reduction of immunosuppression with Tacrolimus. CMV-DNA viral load decreased, but the status of severe leukopenia (760 cells/μL) and thrombocytopenia (126,000 platelet/mm^3^) persisted. Despite immunosuppression reduction. No circulating IFN-γ secreting CMV-specific T-cells were detected in the blood. CMV-DNA was also detected (9700 copies/mL) in a sample of bone marrow biopsy.

When the clinical case was treated and studied in our institution, the use of antiviral drugs, like Foscarnet or Cidofovir, was excluded for treating CMV infection because of potential severe toxicity. In order to understand if the patient developed mutations responsible for conferring GCV resistance, samples at each time point during antiviral treatment (three samples pretreatment, one sample postganciclovir, and one sample postinfusion) were analyzed for the identification of CMV drug-resistance mutations in UL54 (codons 350-859) and UL97 (codons 440-641) genes. No drug resistance mutations were found (data not shown).

Finally, CMV genotyping analysis was performed for two variable sequence regions of the CMV gB gene, able to distinguish gB1, gB2, gB3 and gB4. The presence of both CMV genotype 2 (gB2) and 4 (gB4) was found in all analyzed samples. Considering the three relapses of infection with high level of viral replication after antiviral treatment suspension, severe leukopenia and absence of CMV specific cellular immune response in the patient was detected. Thus an adoptive immunotherapy approach with the use of heterologous CMV-specific T lymphocytes (CTL) was decided. The possible infusion of heterologous CTL was decided considering the high number of HLA (A*02, B*13, DRB1*07) matches (5/6 loci) between mother (donor) and son (recipient), due to the fact that the patient’s parents were first cousins.

The CMV-reactive T-cell preparation from peripheral blood mononuclear cells (PBMCs) of the patient’s mother, and the following adoptive immunotherapy treatment was approved by ISMETT Institutional Research Review Board with the following project identification number: IRRB/32/10 and by ISMETT Institutional Ethic Committee. Written informed consent was obtained from the subjects involved in the study.

## 2. Materials and Methods

### 2.1. Generation of Monocyte-Derived Dendritic Cells (DCs)

PBMCs from a CMV-seropositive donor (recipient’s mother) were isolated from blood using a cell preparation tube with sodium citrate (BD Vacutainer ^®^ CPT™). Monocytes were allowed to adhere in six well plates for 3 h. The nonadherent peripheral blood lymphocytes were removed, and the monocytes were differentiated into DCs in RPMI 1640 medium (Lonza, Basel, Switzerland) supplemented with 10% of autologous serum, 100 ng/mL of interleukin (IL) 4 (R&D, Abingdon, UK), and 100 ng/mL of granulocyte-macrophage colony-stimulating factor, GM-CSF (R&D, Abingdon, UK) for seven days in a 5% carbon dioxide humidified incubator at 37 °C.

Before PBMC stimulation, dendritic cells were pulsed with 10 ng/mL peptide for 2 h at 37 °C and irradiated at 30 Gy. The peptides were a pool of fifteen amino acid peptides (15-mers) overlapping by 11 amino acids spanning the entire IE-1 and pp65 protein sequences (JPT, Berlin, Germany).

### 2.2. Preparation of CMV-Reactive T Cell and Analysis of T-Cell Subsets

CMV-reactive T cells were generated as previously described [23]. Briefly, PBMCs were plated in 24-well tissue culture plates at 2 × 10^6^ cells per well with pulsed dendritic cells (50.000 irradiated cells each wells). At day 8 and 15, cells were restimulated with irradiated PBMCs pulsed with 5 μg/mL CMV antigen pools (pp65 and IE-1 peptides mix) (JPT, Berlin, Germany). From day 8, rIL-2 (Proleukin, Novartis, Basel, Switzerland) was added to the cultures every 2 days to further stimulate T-cell proliferation.

Cell surface phenotype of DCs and T cells was investigated using the following monoclonal antibodies: CD56 PE (clone MY31), CD16 PE (clone B73.1), CD19 FITC (clone HIB19), CD20 PE Cy7 (clone L27), HLA-DR PerCP (Clone L243), TCRγδ FITC (clone 11F2), CD8 APC Cy7 (clone SK1), CD4 PE Cy7(clone SK3), CD3 FITC (clone SK7), CD14 APC H7 (clone MφP9), CD80 FITC (Clone L307.4), CD83 FITC (clone HB15e), CD1a APC (clone HI149), CD40 FITC (clone 5C3) (all from Becton Dickinson, Franklin Lakes, NJ, USA). The cell phenotypes were dissected with a FACS Aria II flow cytometer/Cell sorter and FACS Diva software version 6.1.2. (Becton Dickinson, Franklin Lakes, NJ, USA) was used to analyze the data. Appropriate matched isotype controls (Becton Dickinson, Franklin Lakes, NJ, USA) were used in each assay.

### 2.3. Cytotoxicity Assay

The cytotoxic activity of CMV-CTL was evaluated in a standard 4-h 51Cr release assay [24,25]. Target cells were PBMCs stimulated with three days phytohemagglutinin (PHA) treatment. We tested CMV-specific and nonspecific target cells: (1) autologous donor blasts pulsed with CMV-peptides; (2) autologous donor blasts; (3) recipient pulsed CMV-peptides blasts and (4) recipient blasts. The absence of alloreactivity (<10% cytotoxicity) was assessed using recipient-derived PHA blasts as target cells in a standard 51Cr release assay.

### 2.4. Interferon-γ Detection

Enzyme-Linked ImmunoSPOT (ELISPOT) assays were conducted using the Human Interferon-Gamma Basis-kit from GmBH following the manufacturer’s instructions. Briefly, CTLs (1.5 × 10^5^) or PBMCs (2.5 × 10^6^) were added in combination with 5 × 10^4^ of the following stimulators: (1) autologous donor pulsed CMV-peptides PHA blasts; (2) recipient pulsed CMV-peptides PHA blasts and (3) pp65 and IE-1 peptides mix. The spots of IFN-g producing T cells were counted in ELISPOT reader (Autoimmun Diagnostika, AID, Strasberg, Germany) and negative control values (unstimulated cells) were subtracted from the corresponding stimulated sample values.

### 2.5. Monitoring of CMV-DNA Load

We determined CMV-DNA load by quantitative polymerase chain reaction (PCR) using the standardized and commercially available Q-CMV Real Time Complete Kit (Nanogen Advanced Diagnostics S.r.L., Torino, Italy) on three different whole blood samples. T-cell lines were prepared from fresh samples of peripheral blood of the patient’s mother.

### 2.6. CMV Genotyping Resistance Testing

Drug resistance mutation analysis was performed by sequencing of the UL54 gene (codons 350 to 859), and the UL97 gene (codons 440 to 641), which have been used to identify the most common mutations conferring resistance to antivirals currently identified [26]. CMV DNA was extracted from 200 μL of whole blood using the QIAamp DNA Mini Kit (QIAGEN, Hilden, Germany) following the manufacturer’s instructions. Amplification and sequencing analysis of UL54 and UL97 genes were performed using primers previously reported [27]. DNA sequencing reactions were purified using BigDye XTerminator Purification Kit (Thermo Fisher Scientific, Waltham, MA, USA) and sequencing was performed by capillary electrophoresis in a 3500 Genetic Analyzer (Thermo Fisher Scientific, Waltham, MA, USA). The sequences were analyzed with Sequencing Analysis v5.4 and SeqScape v2.7 software and compared with reference stain AD169 (GenBank accension numbers BK000394 and X17403) to determine the presence of known drug resistance mutations. Moreover, UL54 and UL97 sequences were examined using an online HCMV drug resistance mutations tool (http://www.informatik.uni-ulm.de/ni/mitarbeiter/HKestler/hcmv/ (accessed on 15 May 2014)) [28].

### 2.7. Analysis of CMV Encoded miRNAs

Total RNA was isolated from 500 μL of whole blood by using the RiboPure™-Blood Kit (Thermo Fisher Scientific, Waltham, MA, USA) according to the manufacturer’s instructions. The viral profiling for CMV-miRNA expression was performed using Custom TaqMan^®^ Array MicroRNA Cards (Thermo Fisher Scientific, Waltham, MA, USA), which include 24 CMV miRNA in a 384-well format (assay ID are listed in Supplementary Table S1). In brief, total RNA was first reverse-transcribed with the Multiplex RT pool set (Thermo Fisher Scientific, Waltham, MA, USA) through a reverse transcription (RT) step using the TaqMan^®^ MicroRNA Reverse Transcription Kit (Thermo Fisher Scientific, Waltham, MA, USA). The RT products were subsequently amplified with sequence-specific primers using the Applied Biosystems 7900 HT Real-Time PCR system according to the manufacturing protocol.

### 2.8. Cellular miRNA Profiling

Cell host microRNA profiling of samples was performed using TaqMan Array Human MicroRNA panels A and B (Thermo Fisher Scientific, Waltham, MA, USA) to analyze 754 human miRNAs. Reverse transcription and preamplification were performed following the manufacturer’s instructions (Thermo Fisher Scientific, Waltham, MA, USA). Real-time PCR was performed with the Applied Biosystems 7900 HT Real-Time PCR system.

### 2.9. Data Analysis and Statistical Analysis

Data processing and analysis were done with tools from RQ-manager (v1.2, Life Technologies, Thermo Fisher Scientific), Expression Suite software (v1.0.4, Life Technologies, Thermo Fisher Scientific), Microsoft Excel and Prism GraphPad 8.4.3 software (GraphPad Software, La Jolla, CA, USA, www.graphpad.com (accessed on 25 March 2021)). Hierarchical clustering of miRNA expression data was performed using Euclidean distance algorithms with Cluster 3.0 program and the heat maps were generated using the Java TreeView program.

The Spearman correlation coefficient (*r*) was estimated to determine the linear association between the viral load values and the microRNAs expression variables using a logarithmic scale. The outcome results were interpreted according to the degree of association as strong (*r* = 0.7–1), moderate (*r* = 0.5–0.7), or low (*r* = 0.3–0.5) after taking significant correlation (*p* < 0.01 or *p* < 0.05) values into consideration.

## 3. Results

### 3.1. CMV-Reactive T Cells Generation

PBMCs and autologous DCs from CMV seropositive donor were cocultured for three weeks. The lymphocytes proliferated quickly, and we obtained 80 × 10^6^ cells. Before infusions, cells were tested for sterility, specificity and activity against viral antigens.

The generated cells contained 96% CD3^+^ cells with 74% and 24% CD8^+^ and CD4^+^ cells, respectively. Only 2% of Natural Killer (NK) cells and 0.2% CD19^+^ B cells were detected. Before infusion, IFN-γ-secreting donor T cell analysis revealed a specific response, after stimulation with autologous blasts pulsed with CMV-peptides (187 SFC secreted IFN-γ/millions of cells). The number of SFC for pp65 peptide pool stimulation was 943/millions of cells, while for IE-1 pool was a weaker 38/million cells.

**Figure 1:**
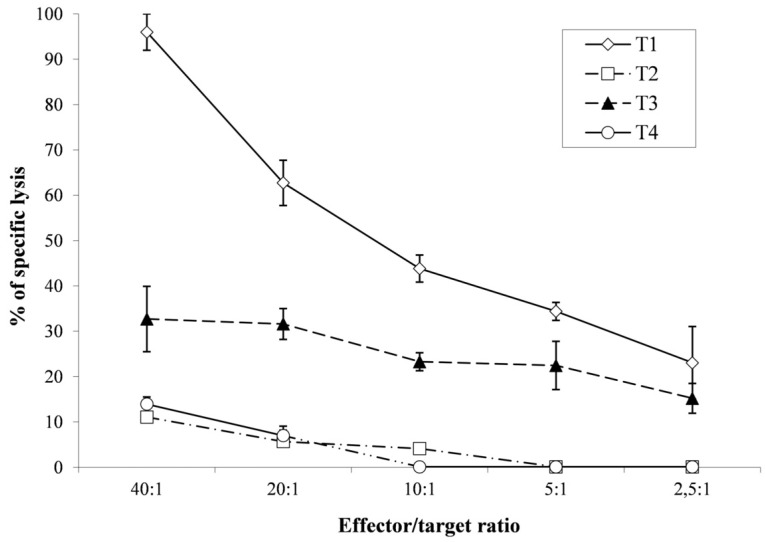
51CR Cytotoxicity assay of T-Lymphocyte clones after three stimulations, T-Lymphocytes killing of specific and nonspecific targets at different ratio. T1 autologous donor blasts pulsed with cytomegalovirus (CMV)-peptides, T2 autologous donor blasts, T3 recipient blasts pulsed CMV-peptides and T4 recipient blasts.

The donor CMV-reactive T lymphocytes killed autologous IE1/pp65 pulsed target cells (60% killing at an effector-to-target ratio of 20:1). Interestingly, the effector cells also showed a good response against recipient IE1/pp65 pulsed recipient target cells (32% of specific lysis at the same ratio). The absence of alloreactivity, using recipient-derived PHA blasts as target cells is shown in Figure 1.

For adoptive transfer, a dose of 10^6^ cells/Kg was infused once, and the second administration was performed 15 days later. Both infusions were well tolerated, and no further adverse events were noted over 12 months of follow-up. One week after the second infusion the leucocytes number increased from 2630 lymphocytes/μL to 6720 cell/μL.

**Figure 2:**
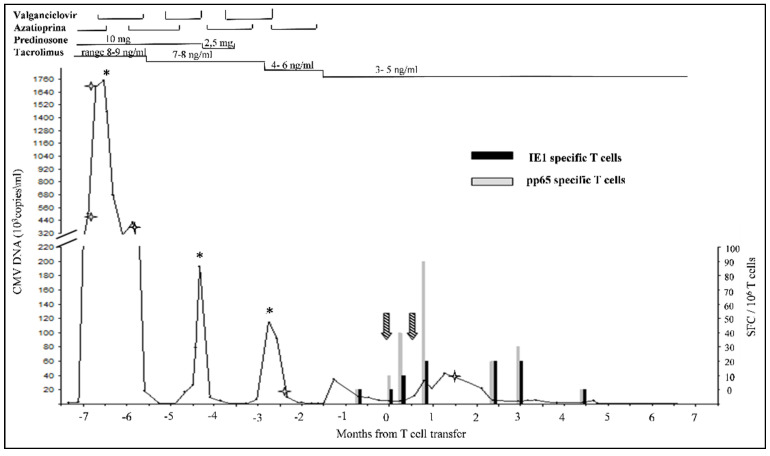
Trends of CMV DNA viral load and IFN-γ secreting T cells following stimulation with peptide mix of IE-1 and pp65 proteins, (SFC: spot forming cells) during 15 months period. Time points of infusion are marked by streaked arrows. The asterisk * indicates GCV administration timepoints. The star 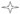 indicates the timepoints of drug resistance mutations analysis. The upper part of the figure shows the immunosuppressant therapy administrated during the timeline.

The post infusion viral load dropped constantly after an initial increase. This might indicate extensive lysis of CMV-infected cells (Figure 2).

The CMV-DNA value in bone marrow was 9700 copies/mL before T cells transfer, and after first infusion it decreased to 823 copies/mL. The patient’s CMV-DNA values were monitored showing a level lower than surveillance cut off and, for the following ten years, no more relapse was recorded.

### 3.2. CMV-Reactive T Cells Recovery

To assess the recovery of CMV-reactive T cells after treatment, we collected recipient PBMCs and tested for IFN-γ secretion by Elispot assay.

Before infusion, the PBMC patient never responded significantly to these stimuli, but one week after the second infusion, as shown in Figure 2, the number of spots corresponding to IFN-γ secreting cells (stimulated with pp65) increased to 90 spots/10^6^ PBMC and after two months the number of IFN-γ secreting cells decreased to zero, probably due to the clearance of donor CMV-reactive T cells.

#### 3.2.1. Host Cellular miRNome Evaluation

CMV has a deep impact on the regulation of host cell metabolism by altering levels of cellular transcripts or through the interaction with host miRNAs affecting their regulation [29]. To have an overview of the impact of HCMV infection on host cellular miRNome, we profiled 754 human cellular miRNAs (Figure S1) from whole blood of the patient across 18 time points covering all the CMV infection/relapses phases.

**Figure 3:**
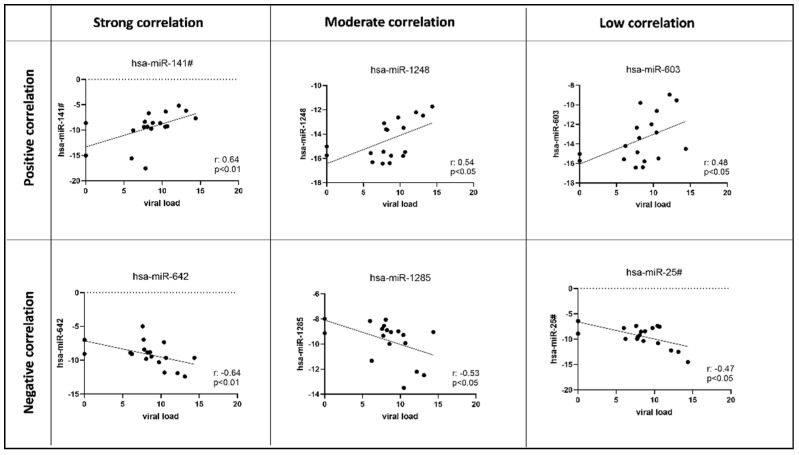
Scatter plot showing statistically significant (two-tailed) Spearman positive (hsa-miR-141#, hsa-miR-1248, hsa-miR-603) and negative (hsa-miR-642, hsa-miR-1285, hsa-miR-25#) microRNAs plasmatic expression levels correlations with CMV DNA (copies/mL).

**Figure 4:**
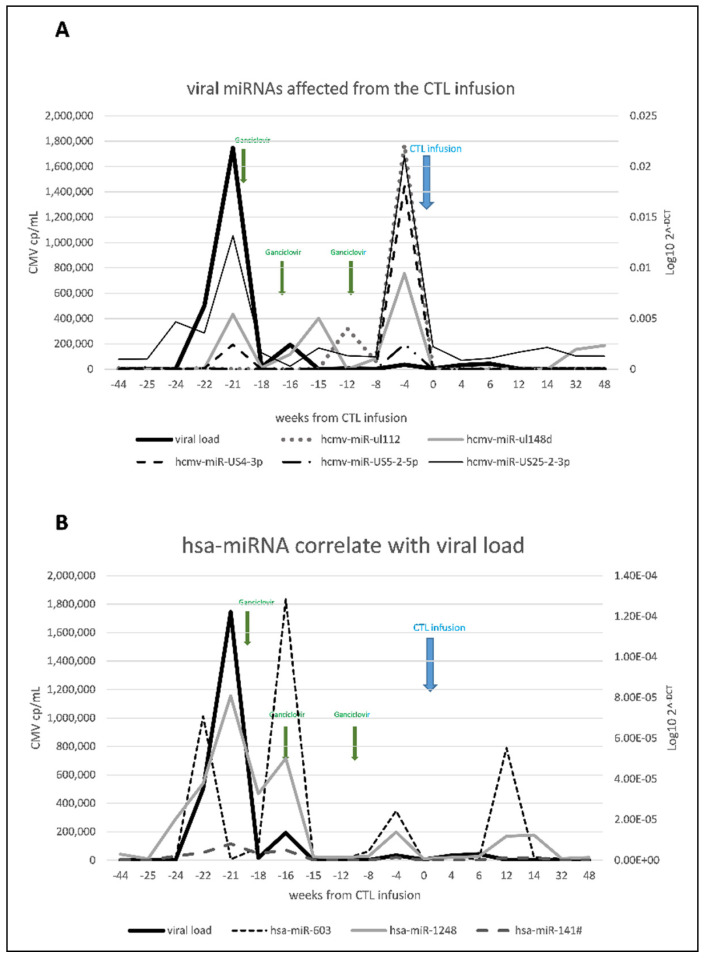
(**A**) Time-course of CMV DNA viral load and the expression profiles of CMV miRNAs, hcmv-miR-ul112, hcmv-miR-ul148d, hcmv-miR-US4-3p, hcmv-miR-US5-2-5p and hcmv-miR-US25-2-3p in relation with the CMV-reactive T-Lymphocyte infusion. Green arrows indicate Ganciclovir (GCV) administration, and the blue arrow indicates the CMV-reactive T-cells infusion. (**B**) Time-course of CMV DNA viral load and the expression profiles of host cellular miRNAs, hsa-miR-603, hsa-miR-1248 and hsa-miR-141#, in relation with the CMV-reactive T-Lymphocyte infusion. Green arrows indicate GCV administration, and the blue arrow indicates the CMV-reactive T-cells infusion.

The analysis showed that the majority of microRNAs maintained a constant expression across the time line, and only few microRNAs were impacted from the CMV infection, in accordance with other previous studies [29]. In order to evaluate a possible response of the host microRNAs to the CMV infection/relapses phases during the CMV 15 month’s infection, we analyzed the array data and correlated their expression with the viral load (Figure 4B). We found that only six of the 754 microRNAs analyzed significantly correlated with the viral load, suggesting that the expression of those microRNAs could have been impacted by the infection. Hsa-miR-141#, hsa-miR-1248 and hsa-miR-603 showed statistically significant positive correlations, while hsa-miR-642, hsa-miR-1285 and hsa-miR-25# negatively correlated with the viral load (Figure 3).

#### 3.2.2. CMV miRNome Evaluation

Considering the relevant modulatory effects of the viral miRNAs on infected cells, we decided to investigate the CMV miRNome circulating in the peripheral blood of the patient for the first 15 months post-transplant. Of the 26 mature miRNA encoded by the CMV genome [30,31], we assessed the expression of 24 miRNA available commercially (Table S1) and detected the expression of the following 11 circulating v-miRNAs: hcmv-miR-ul22a, hcmv-miR-us25-1, hcmv-miR-US29-3p, hcmv-miR-US5-1, hcmv-miR-us25-2-5p, hcmv-miR-ul112, hcmv-miR-ul148d, hcmv-miR-US4-3p, hcmv-miR-US5-1, hcmv-miR-US5-2-5p and hcmv-miR-US25-2-3p (Figure S2).

Interestingly, among them five and, more specifically, hcmv-miR-ul112, hcmv-miR-ul148d, hcmv-miR-US4-3p, hcmv-miR-US5-2-5p and hcmv-miR-US25-2-3p, followed the viral relapses and dropped dramatically only after CMV-reactive T lymphocytes infusion, indicating the transcriptional status of the virus and, perhaps, the end of active viral replication (Figure 4A).

## 4. Discussion

Increasing clinical experience in the management of CMV infection after solid organ transplantation, and the quantification of viral load, allows for rational approaches in defining the cut off levels for the appearance of clinical symptoms and for the initiation of pre-emptive antiviral therapy. Protracted and recurrent antiviral treatment and drug-resistance represent a significant limitation to the therapy. Once patients have failed therapy, the clinical outcome is poor and the use of cellular immunotherapy becomes particularly attractive, as it might prevent and treat virus-related complications.

In this clinical report, we describe treatment of an SOT recipient with severe and persistent CMV infection, by using an allogenic T cell therapy. The patient’s CMV showed no mutations that confer GCV resistance, despite antiviral treatment, and the strategy to decrease immunosuppressive therapy was unable to clear CMV infection. Probably, in the CMV infection detected in bone marrow tissue, severe leukopenia and immunosuppression therapy affected the response to GCV therapy.

To treat the patient, two doses of the patient’s mother’s CMV-reactive T-cells, expanded and activated in vitro, were infused. Allogenic T-lymphocyte generation was evaluated because of the patient’s severe leukopenia and good HLA-matching of donor-recipient, since the recipient’s parents were related by blood. The infusions were well tolerated, and no side effects were observed.

After treatment, there was an increase in virus-responsive IFN-γ producing circulating T cells and, probably, these cells were able to control viral replication successfully.

The patient’s CMV-DNA values were kept to a level lower than surveillance cut off and, in the following ten years, no relapse was recorded. This therapeutic approach in HSCT recipients controls CMV reactivation and disease [32]. Recently, in an SOT recipient, successful use of third-party partially HLA-matched CMV specific T-cells was described in a case report for treatment of refractory or drug-resistant CMV disease [33]. A similar treatment strategy was described in a previous study using allogenic Epstein-Barr-specific T-cell lines to treat post transplantation lymphoproliferative disease in SOT patients [34].

Here, with regard to the miRNA expression profile in the peripheral blood, we checked for cellular miRNAs expression profiles. We found that only six of the 754 microRNAs analyzed significantly correlated with the viral load (positive correlation: hsa-miR-141#, hsa-miR-1248 and hsa-miR-603 and negative correlation: hsa-miR-642, hsa-miR-1285 and hsa-miR-25#) suggesting that the expression of those microRNAs was influenced by the infection status, making them potential biomarkers. We also investigated viral miRNAs, and five of them (hcmv-miR-ul112, hcmv-miR-ul148d, hcmv-miR-US4-3p, hcmv-miR-US5-2-5p and hcmv-miR-US25-2-3p) followed the viral relapses and dropped dramatically only after the CMV-reactive T lymphocyte infusion, suggesting their role in tracking an active replication status in response to CMV-reactive T-cell infusion.

This case report showed that an immunotherapy approach, by generation and infusion of allogenic T-cells, could be used to treat CMV infections when the production of autologous effector cells is not possible. This therapy is a useful tool for viral-specific immune reconstitution in transplanted patients and appears to be associated with clinical protection from recurrent infection.

Therefore, this approach is an interesting area for future clinical applications due to the absence of side effects and could provide a valid alternative to the use of antiviral drugs.

## Supplementary Materials

The following are available online at https://www.mdpi.com/article/10.3390/microorganisms9040684/s1, Figure S1: Unsupervised hierarchical clustering of the time-course expression host miRNA profiles; Figure S2: Unsupervised hierarchical clustering of the time-course expression viral miRNA profiles; Table S1: Assay IDs for CMV miRNAs.
