# Supplementary material for: Successful Use of Heterologous CMV-Reactive T Lymphocyte to Treat Severe Refractory Cytomegalovirus (CMV) Infection in a Liver Transplanted Patient: Correlation of the Host Antiviral Immune Reconstitution with CMV Viral Load and CMV miRNome
Source: Microorganisms. 2021 Mar 26;9(4):684. doi: 10.3390/microorganisms9040684 (PMC8066103; doi:10.3390/microorganisms9040684)
Supplement: Supplementary file 1 [file microorganisms-09-00684-s001.zip › Supplementary Table I.pdf]

Supplementary Table I. Assay IDs for CMV miRNAs.

**Assay Name**	**Assay ID**
hcmv-miR-us25-2-5p	7201_mat
hcmv-miR-ul22a*	6040
hcmv-miR-UL112-5p	469687_mat
hcmv-miR-us25-1	197211_mat
hcmv-miR-US5-2-3p	469255_mat
hcmv-miR-ul36	197212_mat
hcmv-miR-US4-5p	469977_mat
hcmv-miR-US5-2-5p	469274_mat
hcmv-miR-US4-3p	469699_mat
hcmv-miR-US22-5p	468736_mat
hcmv-miR-ul112	6621
hcmv-miR-ul148d	197215_mat
hcmv-miR-US5-1	004641_mat
hcmv-miR-UL70-5p	003183_mat
hcmv-miR-US22-3p	468548_mat
hcmv-miR-ul22a	7677
hcmv-miR-US25-2-3p	468261_mat
hcmv-miR-UL69	468621_mat
hcmv-miR-ul36*	197227_mat
hcmv-miR-US33-3p	468703_mat
hcmv-miR-UL59	6481
hcmv-miR-US25-1-3p	468308_mat
hcmv-miR-US29-3p	468621_mat
hcmv-miR-us33-5p	197227_mat
