# Supplementary figures and images for: Successful Use of Heterologous CMV-Reactive T Lymphocyte to Treat Severe Refractory Cytomegalovirus (CMV) Infection in a Liver Transplanted Patient: Correlation of the Host Antiviral Immune Reconstitution with CMV Viral Load and CMV miRNome
Source: Microorganisms. 2021 Mar 26;9(4):684. doi: 10.3390/microorganisms9040684 (PMC8066103; doi:10.3390/microorganisms9040684)

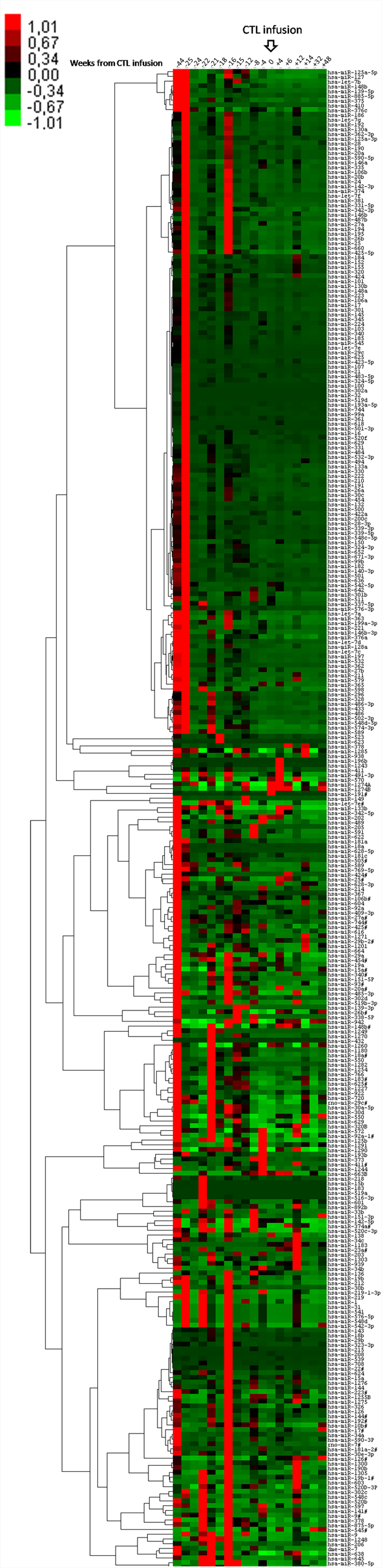

Supplement: Supplementary file 1 [file microorganisms-09-00684-s001.zip › Figure S1.tif]

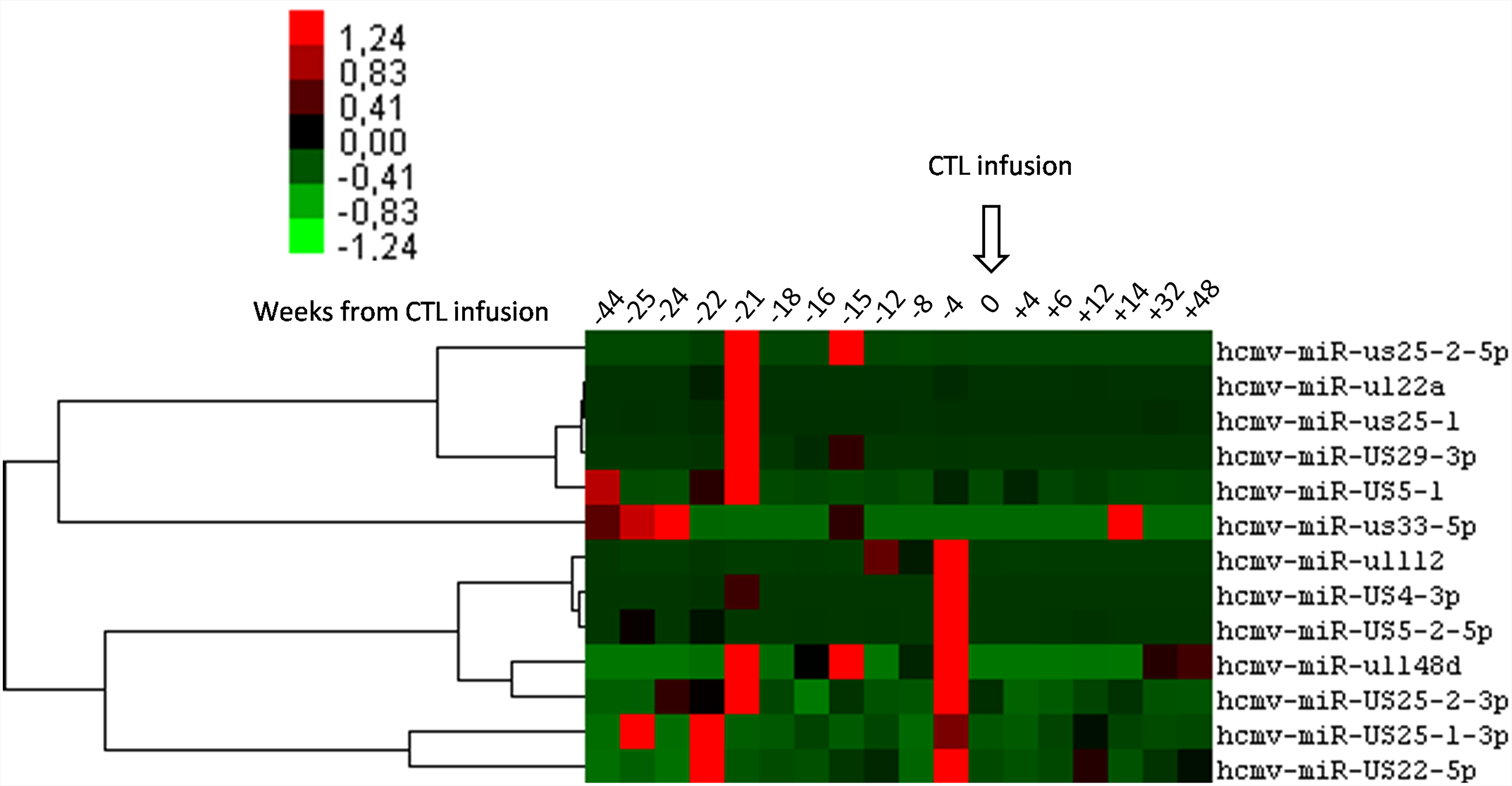

Supplement: Supplementary file 1 [file microorganisms-09-00684-s001.zip › Figure S2.tif]
